# Gene expression and activity of cartilage degrading glycosidases in human rheumatoid arthritis and osteoarthritis synovial fibroblasts

**DOI:** 10.1186/ar2697

**Published:** 2009-05-14

**Authors:** Mária Pásztói, György Nagy, Pál Géher, Tamás Lakatos, Kálmán Tóth, Károly Wellinger, Péter Pócza, Bence György, Marianna C Holub, Ágnes Kittel, Krisztina Pálóczy, Mercédesz Mazán, Péter Nyirkos, András Falus, Edit I Buzas

**Affiliations:** 1 Department of Genetics, Cell and Immunobiology, Semmelweis University, Nagyvárad tér 4, Budapest H-1089, Hungary; 2 Department of Rheumatology, Semmelweis University, Frankel Leó utca 54, Budapest H-1027, Hungary; 3 Department of Orthopedic Surgery, Szeged University, Semmelweis u.6, Szeged H-6725, Hungary; 4 Institute of Experimental Medicine, Hungarian Academy of Sciences, Szigony u. 43, Budapest H-1083, Hungary; 5 Inflammation Biology and Immunogenomics Research Group, Hungarian Academy of Sciences-Semmelweis University, Nagyvárad tér 4, Budapest H-1089, Hungary

## Abstract

**Introduction:**

Similar to matrix metalloproteinases, glycosidases also play a major role in cartilage degradation. Carbohydrate cleavage products, generated by these latter enzymes, are released from degrading cartilage during arthritis. Some of the cleavage products (such as hyaluronate oligosaccharides) have been shown to bind to Toll-like receptors and provide endogenous danger signals, while others (like *N*-acetyl glucosamine) are reported to have chondroprotective functions. In the current study for the first time we systematically investigated the expression of glycosidases within the joints.

**Methods:**

Expressions of β-D-hexosaminidase, β-D-glucuronidase, hyaluronidase, sperm adhesion molecule 1 and klotho genes were measured in synovial fibroblasts and synovial membrane samples of patients with rheumatoid arthritis and osteoarthritis by real-time PCR. β-D-Glucuronidase, β-D-glucosaminidase and β-D-galactosaminidase activities were characterized using chromogenic or fluorogenic substrates. Synovial fibroblast-derived microvesicles were also tested for glycosidase activity.

**Results:**

According to our data, β-D-hexosaminidase, β-D-glucuronidase, hyaluronidase, and klotho are expressed in the synovial membrane. Hexosaminidase is the major glycosidase expressed within the joints, and it is primarily produced by synovial fibroblasts. HexA subunit gene, one of the two genes encoding for the alpha or the beta chains of hexosaminidase, was characterized by the strongest gene expression. It was followed by the expression of HexB subunit gene and the β-D-glucuronidase gene, while the expression of hyaluronidase-1 gene and the klotho gene was rather low in both synovial fibroblasts and synovial membrane samples. Tumor growth factor-β1 profoundly downregulated glycosidase expression in both rheumatoid arthritis and osteoarthritis derived synovial fibroblasts. In addition, expression of cartilage-degrading glycosidases was moderately downregulated by proinflammatory cytokines including TNFα, IL-1β and IL-17.

**Conclusions:**

According to our present data, glycosidases expressed by synovial membranes and synovial fibroblasts are under negative regulation by some locally expressed cytokines both in rheumatoid arthritis and osteoarthritis. This does not exclude the possibility that these enzymes may contribute significantly to cartilage degradation in both joint diseases if acting in collaboration with the differentially upregulated proteases to deplete cartilage in glycosaminoglycans.

## Introduction

Rheumatoid arthritis (RA) is a chronic, progressive systemic autoimmune disease that affects approximately 1% of the adult population. Proinflammatory cytokines and chemokines are considered to be the key regulators, and certain proteases to be the major effector molecules, in the pathomechanism of the disease.

There has been a recent increasing awareness of the significance of post-translational protein modifications in health and disease. In rheumatology this is best exemplified by the significance of citrullination [1-3]. Even though glycosylation is the most frequent post-translational modification, its role is still poorly understood. Enzymes that collaborate to determine the final structures of glycans are glycosyl transferases and glycosidases. The significance of glycosidases has been recently suggested by studies in which glycosidase activity resulted in abrogation of arthritogenicity of IgG [4]. The current study focuses on glycosidases expressed locally, within the joints.

Earlier we found very low enzyme activities of α-D-mannosidase and β-D-galactosidase in serum and synovial fluid (SFl) of patients with RA and osteoarthritis (OA). On the contrary, SFl exoglycosidases (β-D-*N*-acetyl-glucosaminidase (NAG) and β-D-glucuronidase (GusB) were characterized by significantly elevated enzyme activities in patients with RA as compared with OA [5]. The NAG and GusB enzymes alone or in combination with matrix metalloproteinases (MMPs) were efficient in degrading hyaline cartilage directly [5]. The measured NAG activity is characteristic for hexosaminidase, the enzyme responsible for the hydrolysis of terminal nonreducing *N*-acetyl-D-hexosamine.

Until recently, β-D-glucuronidase activity was attributed solely to the lysosomal GusB enzyme. The anti-ageing klotho protein, however, was also shown to have β-D-glucuronidase activity [6]. Until now no study had investigated the expression of the klotho gene in synovial fibroblasts (SFs) and synovial membranes (SMs), and neither were any data available on the expression of the hyaluronidase 1 (Hyal1) and sperm adhesion molecule 1 (Spam1) hyaluronidase genes in the joints.

We also extended this work to the glycosidase-like Hc-gp 39 that we discovered earlier as one of the most abundant proteins synthesized by SFs [7]. Hc-gp 39 is classified as a member of the chitinase-like family 18 of proteins because of its amino acid sequence, although no glycohydrolase activity of this molecule has so far been demonstrated [8].

Cell-derived membrane-bound microvesicles (MVs) have also been shown to play an important role in mediating cell – cell communication and in the pathogenesis of several autoimmune diseases [9-13]. Lymphocyte-derived microvesicles activate SFs in a dose-dependent manner to release MMPs, proinflammatory cytokines and chemokines [13].

There is increasing evidence that SFs are key players in the pathogenesis of RA by invading and eroding hyaline cartilage. SFs, activated locally, produce a variety of cytokines, chemokines and matrix-degrading enzymes [14].

In the present work we investigated the effect of paramount cytokines including TNFα IL-1β, IL-17, tumor growth factor beta 1 (TGF-β_1_) and we also studied MVs as potential sources of glycosidases.

The current study describes for the first time the glycosidase expression profile of SFs in RA and OA, and demonstrates that glycosidases are under negative regulation in SFs.

## Materials and methods

### Patients

SFl samples were obtained from the knee joints of 31 patients (six males, 25 females) with RA and of 16 patients (four males, 12 females) with OA treated in the Hospital of Hospitaller Brothers of St John of God, Budapest, Hungary. All the patients suffered from exudative synovitis.

SMs were obtained at joint replacement surgery (in the Hospital of Hospitaller Brothers of St John of God, Budapest and the Department of Orthopedics, University Medical School of Szeged, Hungary) from 10 RA patients (one male, nine females; mean ± standard error mean (range) age, 61.5 ± 10.3 (25 to 79) years) and from 17 OA patients (seven males, 10 females; age, 64.53 ± 7.32 (39 to 79) years). All RA and OA patients met the American College of Rheumatology criteria for RA [15] and for OA [16], respectively.

RA patients were characterized by an erythrocyte sedimentation rate (mean ± standard error mean) of 28.60 ± 18.04 mm/hour, as opposed to 19.00 ± 9.88 mm/hour for patients with OA. The mean C-reactive protein level of RA patients was 22.16 ± 18.85 mg/l, but the C-reactive protein values of OA patients were not determined. The white blood cell count of patients with RA was 8,020 ± 1,360/μl, as compared with 7,019 ± 1,320/μl for patients with OA. The mean ± standard error mean (range) disease duration from diagnosis of RA patients was 10.4 ± 8.4 (0 to 35) years, as compared with 3.5 ± 2.25 (1 to 10) years for OA patients. Medication of RA patients included *per os *methotrexate, methylprednisolone and sulphasalazine.

The study was approved by the Human Investigation Review Board of the University of Szeged and all patients signed an informed consent form.

### Isolation and culture of synovial fibroblasts

SFs were obtained by enzymatic digestion as described by Neidhart and colleagues [17]. Cells were grown in DMEM (Sigma-Aldrich Corp, St. Louis, MO, USA) with 10% FCS (GibcoBRL, Frederick, MD, USA). SFs were cultured for six to eight passages. The cell viability was higher than 95% in all experiments. We found that the repeated passages ensured the purity of fibroblast cell populations without contaminating macrophages, as demonstrated by the lack of staining for CD68 (anti-human CD68-FITC; eBioScience Inc, San Diego, CA, USA). To rule out the possibility that SFs might have changed their native expression profile, we tested baseline glycosidase expression at every second passage, and did not find significant alterations from the P1 to P9 passages either in OA or RA SFs (see Additional data file 1). Gene expression pattern of RA samples may also vary depending on the disease stage. We did not, however, test synovial tissue samples from patients with early-stage RA in the present study.

### Quantitative RT-PCR

Total RNA was extracted from SFs and SMs using the RNeasy^® ^Mini Kit (Qiagen USA, Valencia, CA, USA). Relative quantification of hexosaminidase A subunit (HexA), hexosaminidase B subunit (HexB), GusB, Hyal1, Hc-gp 39, klotho, Spam1, MMP1 and MMP3 mRNAs (referred to mRNA of hypoxanthine phosphoribosyl transferase) was performed with TaqMan quantitative-PCR assays (Hs00166843_m1, Hs00166864_m1, Hs99999908_m1, Hs00537920_g1, Hs00609691_m1, Hs00183100_m1, Hs01095939_m1, Hs00899658:m1 and Hs00233962_m1 referred to Hs99999909_m1, respectively) on an ABI PRISM 7000 Sequence Detector (Applied Biosystems, Foster City, CA, USA) using standard protocols [18].

### Enzyme assays

SMs were homogenized in a Heidolph Diax-type homogenizer on ice in buffer containing 0.2 M phenylmethanesulphonylfluoride, 1 mg/ml PepstatinA, 0.2 M IodoAcetamid, 0.2 M ethylenediamine tetraacetic acid (all purchased from Sigma-Aldrich). SFs were lysed with five freeze – thaw cycles. Enzyme activities were normalized to protein content (50 μg protein was used from all samples) measured by a standard Bradford protein assay. Enzyme activities were measured as described previously [5] and were expressed as units, determined using enzymes with known activities: GUS (EC 3.2.1.31) and NAG (EC 3.2.1.52) (all from Sigma-Aldrich).

### Effect of cytokines on expression and secretion of glycosidases by synovial fibroblasts

SFs were cultured in the presence of human TNFα (BD Biosciences Pharmingen, San Jose, CA, USA), IL-1β and TGF-β_1 _(both from ImmunoTools, Friesoythe, Germany) in 0, 1, 10 and 50 ng/ml concentrations, and of IL-17 (ImmunoTools) in 0, 1, 10 and 100 ng/ml concentrations for 24 hours. The nitric oxide donor (*Z*)-1-(2-(2-aminoethyl)-*N*-(2-ammonioethyl) amino)diazen-1-ium-1,2-diolate diethylenetriamine (NOC-18) (Molecular Probes, Inc., Eugene, OR, USA) was used in 100 and 1,000 μM concentrations. For enzyme release assays, 5 × 10^4 ^cells were cultured in 96-well plates in phenol-red-free-RPMI without FCS in the presence of human TGF-β_1 _for 24 hours. The enzyme activity of both the supernatants and the cell lysates was determined as described above.

### Enzyme histochemistry

SFs were plated onto chamber slides (Nunc Inc., Naperville, IL, USA) and were cultured for 24 hours. Cells were incubated with either 50 μM ImaGene Green C_12_FDGlcU β-D-glucuronidase or ELF^® ^97 *N*-acetylglucosaminide substrates (both from Molecular Probes). The slides were analyzed in a Bio-Rad MRC 1024 confocal laser scanning microscope equipped with a krypton/argon mixed gas laser as the light source (Bio-Rad, Richmond, CA, USA).

### Flow cytometric analysis of synovial fibroblast-derived microvesicles

The SFs were plated at 3 × 10^6 ^cells/75 cm^2 ^flasks in serum-free DMEM. After 24 hours the cell culture supernatants were collected and the spontaneously released MVs were tested immediately. First the supernatant was centrifuged at 500 × *g *for 10 minutes to remove cells, and was then incubated either with 50 μM ImaGene Green C12FDGlcU (the fluorogenic lipophilic substrate of β-D-glucuronidase) or ELF^® ^97 *N*-acetylglucosaminide substrate (both from Molecular Probes) for 30 minutes. To verify the specificity of the reaction, D-glucaric acid-1,4-lactone, a β-D-glucuronidase inhibitor, was used (Molecular Probes). The number of stained MVs was determined by measuring the events for 30 seconds by a FACSCalibur (Beckton Dickinson & Co., San Jose, CA, USA) flow cytometer.

### Electron microscopy of synovial fibroblast-derived microvesicles

SF 24-hour supernatants were centrifuged at 500 × *g *for 10 minutes, and were submitted to ultracentrifugation at 100,000 × *g *for 30 minutes. The pellet was fixed with 2% paraformaldehyde/2% glutharaldehyde for 2 hours, postfixed in 1% OsO_4 _for 30 minutes. The MVs were dehydrated in graded ethanol, block-stained with 2% uranyl acetate in 70% ethanol for 1 hour, and embedded in Taab 812 (Emmer Green, Reading, UK). Ultrathin sections were examined in a Hitachi 7100 transmission electron microscope (Hitachi, Tokyo, Japan).

### Statistical analysis

Statistical analysis was performed using STATISTICA 7.1 (StatSoft Inc. Tulsa, OK, USA).

The Mann – Whitney rank sum test was performed for nonrelated samples and the paired *t *test was used for cytokine-treated samples (after a normality test was passed).

## Results

### Gene expression analysis

First, we analyzed the gene expression of glycosidases including HexA, HexB, GusB, Hyal1, klotho and Hc-gp39 by quantitative PCR. Gene expressions were characterized in SFs and in SMs from RA patients and OA patients.

**Figure 1:**
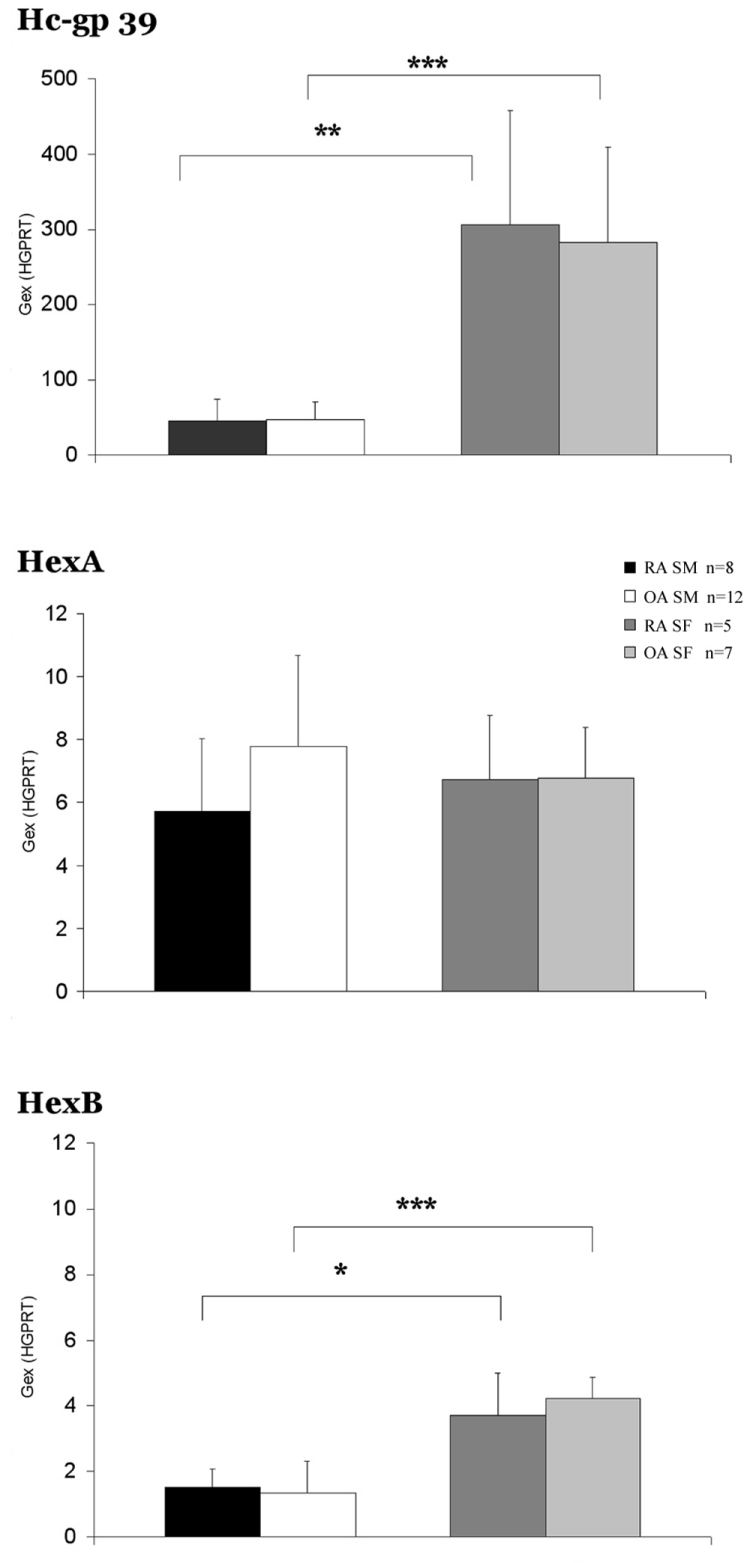
Hc-gp 39, HexA and HexB gene expression in arthritis patients' synovial membrane and fibroblast samples. Rheumatoid arthritis (RA) and osteoarthritis (OA) synovial fibroblasts (SFs) have significantly higher Hc-gp 39 gene expression as compared with RA and OA synovial membranes (SMs) (Mann – Whitney rank sum test). Hexosaminidase A subunit (HexA) gene expression was approximately the same in SFs and in SM tissue samples. Hexosaminidase B subunit (HexB) gene was characterized by significantly higher gene expression in RA and OA SFs as compared with RA and OA SM tissue samples (Mann – Whitney rank sum test). Gex, gene expression; HGPRT, hypoxanthine phosphoribosyl transferase. *P < 0.05, **P < 0.01, ***P < 0.001.

Gene expression of Hc-gp 39 was orders of magnitude higher than that of any of the other tested genes (Figure 1). We have found about 10-fold higher expression of Hc-gp 39 in SFs as compared with SM samples.

HexA and HexB genes were characterized by the second strongest gene expression in all samples (Figure 1). The expression of HexA gene was approximately the same both in SFs and in SMs. In contrast, we observed a significantly higher expression of HexB gene in RA and OA SFs as compared with the SMs. The expression of HexA gene has a tendency to be higher than that of HexB in SFs of RA fibroblasts. In SM samples, however, the dominance of HexA gene expression over HexB was highly significant.

**Figure 2:**
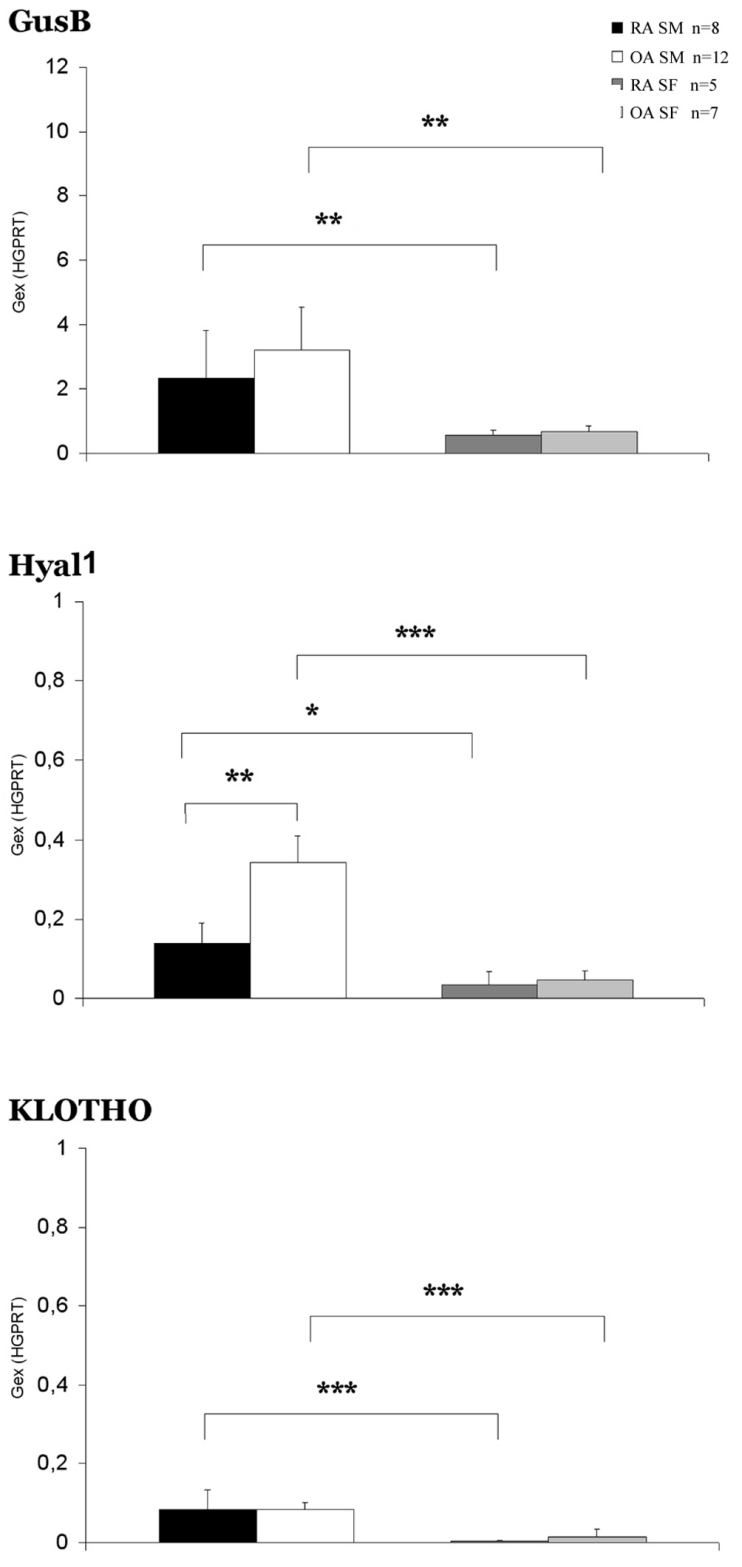
GusB, Hyal1 and klotho gene expression in arthritis patients' synovial membrane and fibroblast samples. Gene expression for β-D-glucuronidase (GusB), hyaluronidase 1 (Hyal1), and klotho genes was lower in rheumatoid arthritis (RA) and osteoarthritis (OA) synovial fibroblasts (SFs) than in RA and OA synovial membrane (SM) tissue samples (Mann-Whitney rank sum test). The Hyal1 gene expression was significantly higher in OA SM as compared with RA SM (Mann – Whitney rank sum test). The sperm adhesion molecule 1 gene expression was undetectable. Gex, gene expression; HGPRT, hypoxanthine phosphoribosyl transferase. *P < 0.05; **P < 0.01; ***P < 0.001.

The expression of GusB, Hyal1 and klotho showed a decreasing sequence of order, as shown in Figure 2. We observed significantly lower expression of these three genes in RA and OA SFs as compared with that in the RA and OA SMs. In OA SMs we found significantly higher Hyal1 expression as compared with the RA SMs. The expression of Spam1 gene was undetectable in any of the samples.

### Enzyme assays

**Figure 3:**
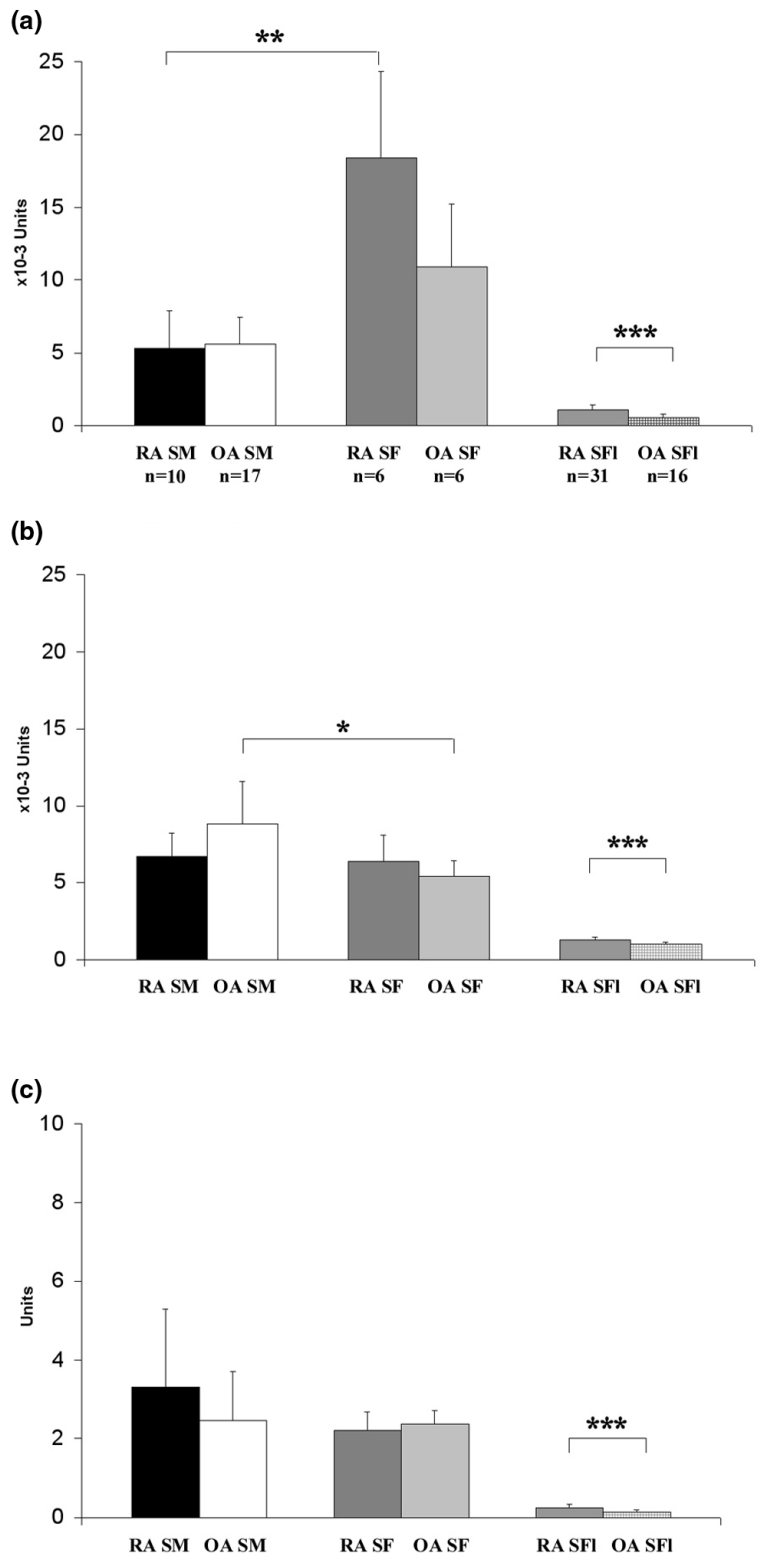
Enzyme activities of synovial membrane, synovial fibroblast and synovial fluid samples from arthritis patients. To determine enzyme activity, the following chromogenic substrates were used: **(a) **β-D-N-acetyl-glucosaminidase, **(b) **β-D-*N*-acetyl-galactosaminidase and **(c) **β-D-glucuronidase. Optical densities were measured at 405 nm. Rheumatoid arthritis (RA) synovial fluid (SFl) showed significantly higher enzyme activities for all tested enzymes as compared with osteoarthritis (OA) SFl. Synovial membrane (SM) and synovial fibroblast (SF) homogenates were characterized by significantly higher enzyme activities as compared with SFl samples. SFs showed significantly higher β-D-*N*-acetyl-glucosaminidase and lower or approximately the same β-D-*N*-acetyl-galactosaminidase and β-D-glucuronidase enzyme activity as compared with SM samples. *P < 0.05; **P < 0.01; ***P < 0.001.

Enzyme activities were measured in SFs, SMs and SFls using chromogenic substrates of NAG, β-D-*N*-acetyl-galactosaminidase and β-D-glucuronidase. The data are summarized in Figure 3.

Activities of NAG, β-D-*N*-acetyl-galactosaminidase and GusB in RA SFls were significantly higher than in OA SFls. The activities of these enzymes in the SFls, however, were markedly lower, quite uniformly, than those detected in the homogenates of either the SMs or the SFs (Figure 3a to 3c). The activity of NAG in RA SFs was significantly higher than in RA SMs. In contrast, the activity of GusB in SFs was lower than in SMs. There was no significant difference in the GusB activities associated with the SM and SF of OA and RA patients (Figure 3a to 3c).

### Detection of GusB and NAG in synovial fibroblasts using lipophilic fluorogenic substrates

**Figure 4:**
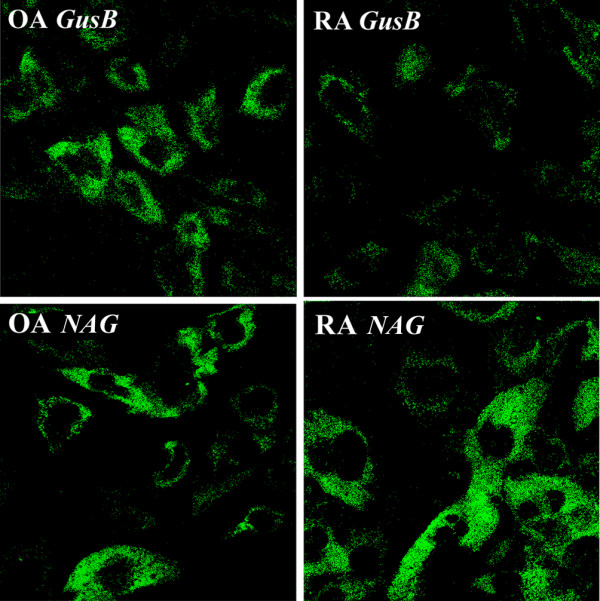
Enzyme-histochemical detection of glycosidases in synovial fibroblast cells. Enzyme-histochemical staining of rheumatoid arthritis (RA) and osteoarthritis (OA) synovial fibroblast monolayers for β-D-glucuronidase (GusB) and β-D-*N*-acetyl-glucosaminidase (NAG) using fluorogenic substrates. Nuclear areas show no fluorescent staining.

RA and OA SF monolayers were stained for GusB and NAG using fluorogenic substrates. Both enzymes are localized to the lysosomes. The intensity of GusB substrate fluorescence was stronger in OA fibroblasts as compared with those isolated from patients with RA (Figure 4). The NAG substrate fluorescence intensity was much higher than that of the β-D-glucuronidase. The NAG staining was more intense in RA fibroblasts as compared with those isolated from OA patients (Figure 4).

### Effect of cytokines and nitric oxide on expression and secretion of glycosidases by synovial fibroblasts

We tested the effect of various cytokines and nitric oxide on the gene expression of glycosidases. Relative gene expression (referred to hypoxanthine phosphoribosyl transferase) was determined by quantitative PCR. The relative gene expression in the unstimulated cells for each gene was defined as 100%.

**Figure 5:**
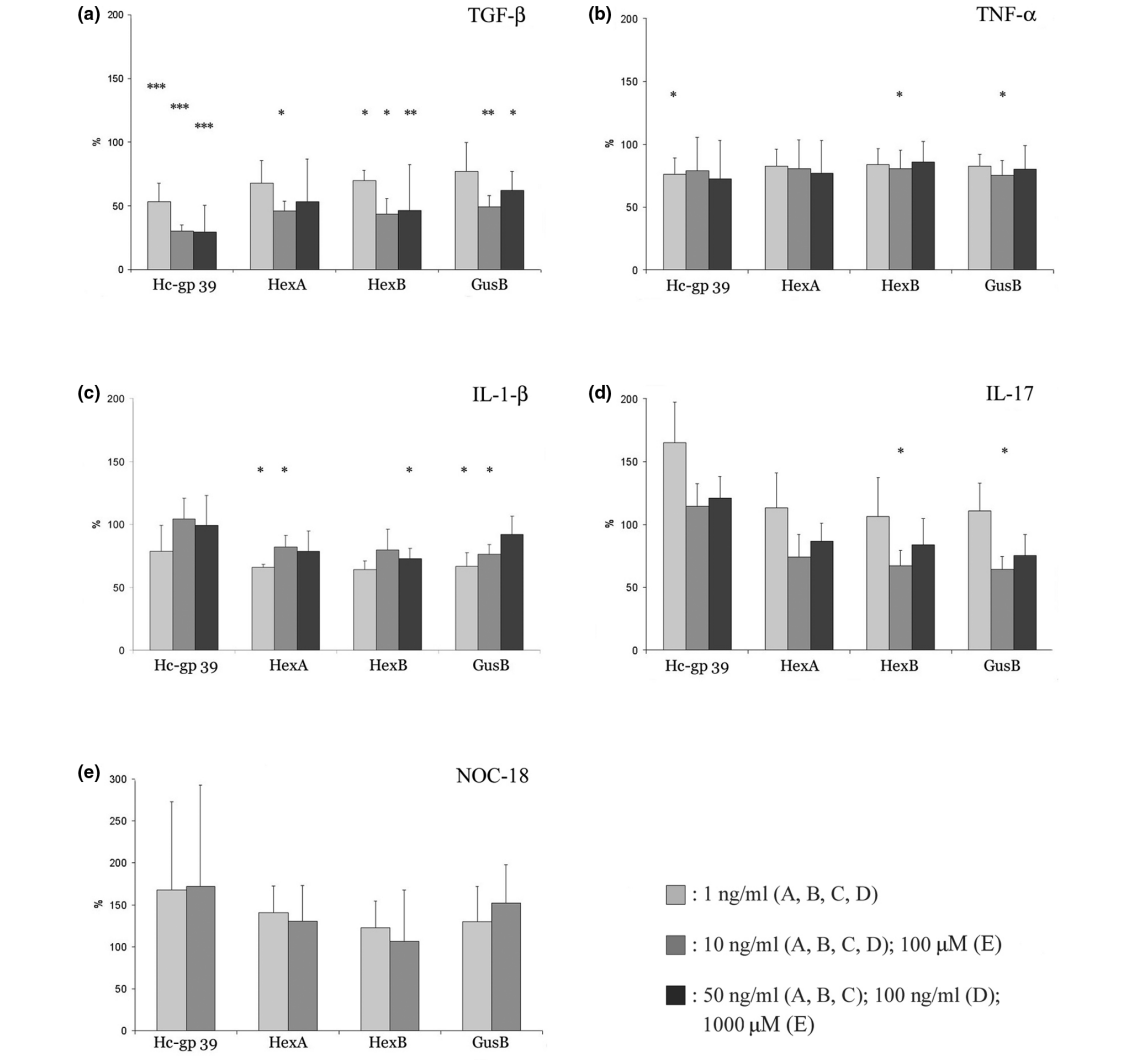
Gene expression of synovial fibroblast samples from rheumatoid arthritis patients after cytokine and NOC-18 treatment. Synovial fibroblasts (SFs) from patients with rheumatoid arthritis (RA) were cultured in the presence or absence of various cytokines or the nitric oxide donor (*Z*)-1-(2-(2-aminoethyl)-*N*-(2-ammonioethyl) amino)diazen-1-ium-1,2-diolate diethylenetriamine (NOC-18) for 24 hours. Relative gene expression (referred to hypoxanthine phosphoribosyl transferase) was determined by realtime PCR. The relative gene expression in the unstimulated cells for each gene is defined as 100%. **(a) **Tumor growth factor beta 1 (TGF-β_1_) stimulation (n = 4). **(b) **TNFα stimulation (n = 6). **(c) **IL-1β stimulation (n = 4). **(d) **IL-17 stimulation (n = 4). **(e) **NOC-18 stimulation (n = 3). Data shown as mean ± standard error mean. *P < 0.05, **P < 0.01, ***P < 0.0015 (paired *t *test). GusB, β-D-glucuronidase; HexA, hexosaminidase A subunit; HexB, hexosaminidase B subunit.

**Figure 6:**
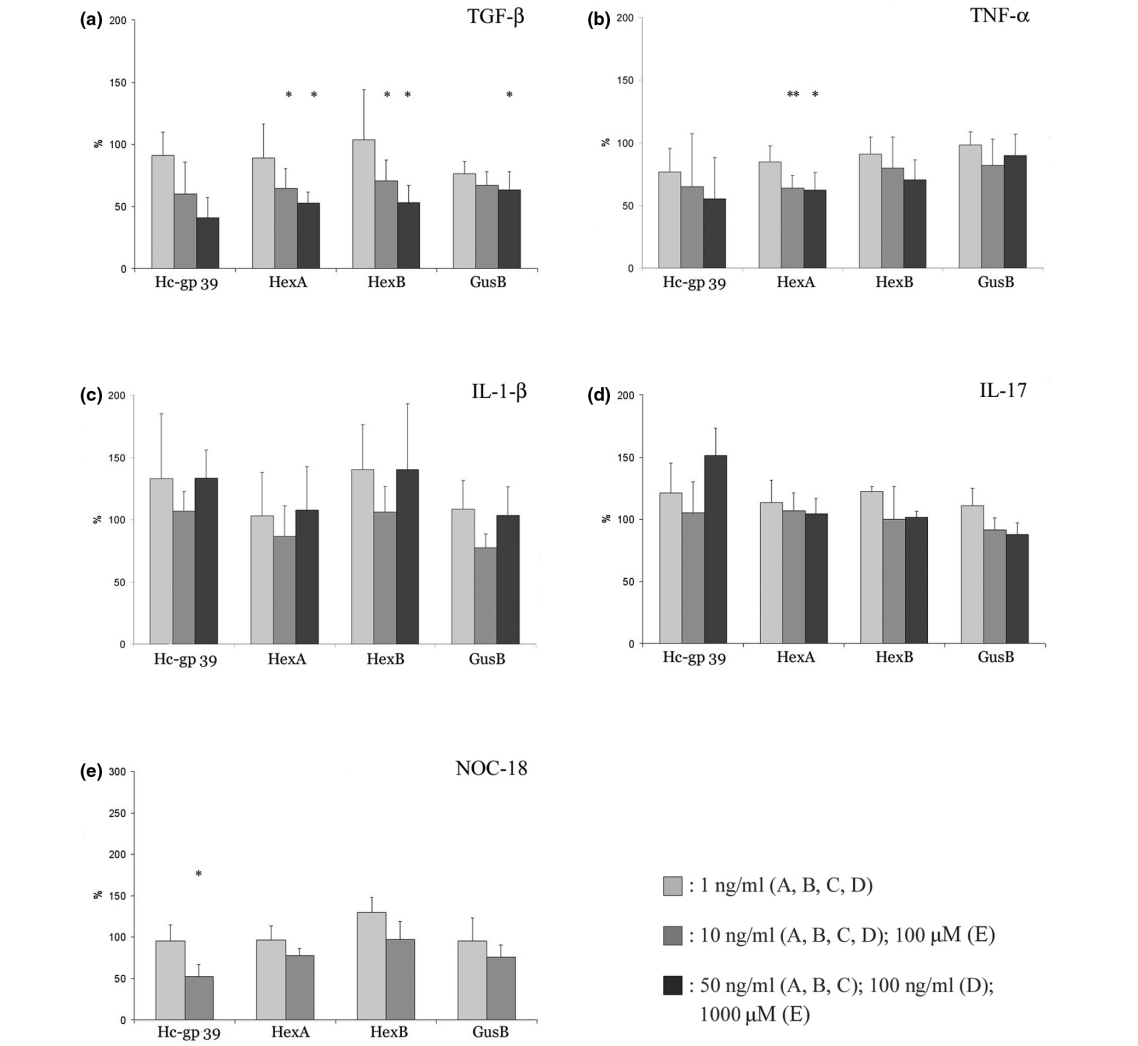
Gene expression of synovial fibroblast samples from osteoarthritis patients after cytokine and NOC-18 treatment. Synovial fibroblasts (SFs) from patients with osteoarthritis (OA) were cultured in the presence or absence of various cytokines or the nitric oxide donor (*Z*)-1-(2-(2-aminoethyl)-*N*-(2-ammonioethyl) amino)diazen-1-ium-1,2-diolate diethylenetriamine (NOC-18) for 24 hours. Relative gene expression (referred to hypoxanthine phosphoribosyl transferase) was determined by realtime PCR. The relative gene expression in the unstimulated cells for each gene is defined as 100%. **(a) **Tumor growth factor beta 1 (TGF-β_1_) stimulation (n = 6). **(b) **TNFα stimulation (n = 6). **(c) **IL-1β stimulation (n = 3). **(d) **IL-17 stimulation (n = 3). **(e) **NOC-18 stimulation (n = 3). Data shown as mean ± standard error mean. *P < 0.05, **P < 0.01, ***P < 0.0015 (paired *t *test). GusB, β-D-glucuronidase; HexA, hexosaminidase A subunit; HexB, hexosaminidase B subunit.

As shown in Figures 5a and 6a, TGF-β_1 _has significantly downregulated the expression of HexA and HexB genes, as well as of GusB and Hc-gp 39. The suppression of gene expression was more pronounced in RA than OA samples (Figures 5a and 6a), and the strongest dose-dependent downregulation was observed in the case of Hc-gp 39 gene. TNFα downregulated the expression of Hc-gp-39, HexB and GusB in RA (Figure 5b), and the expression of HexA gene in OA (Figure 6b). IL-1β significantly decreased the expression of HexA, HexB and GusB in RA (Figure 5c), while it had no effect on gene expression in OA (Figure 6c). The next cytokine tested was IL-17. As shown in Figures 5d and 6d, stimulation of cells by IL-17 in RA decreased the gene expression of both HexB and GusB, whereas in OA it did not have an effect. Finally, we were interested in whether gene expression of the glycosidases was influenced by nitric oxide. In the presence of NOC-18 there was no change in the gene expression, except for Hc-gp 39 being downregulated in OA (Figures 5e and 6e). The expression of Hyal1 was significantly downregulated by TGF-β_1 _(50 ng/ml) and by IL-17 (10 ng/ml) in patients with RA. The gene expression of Hyal1 was very low, however, in all experiments (data not shown).

As a positive control for our assays, we also tested the expression of MMP1 and MMP3 upon stimulation by various cytokines. In all RA SFs, the cytokine-induced upregulation of gene expression of MMP3 was higher than fourfold. NOC-18 did not, however, induce changes in the MMP expression (data not shown).

**Figure 7:**
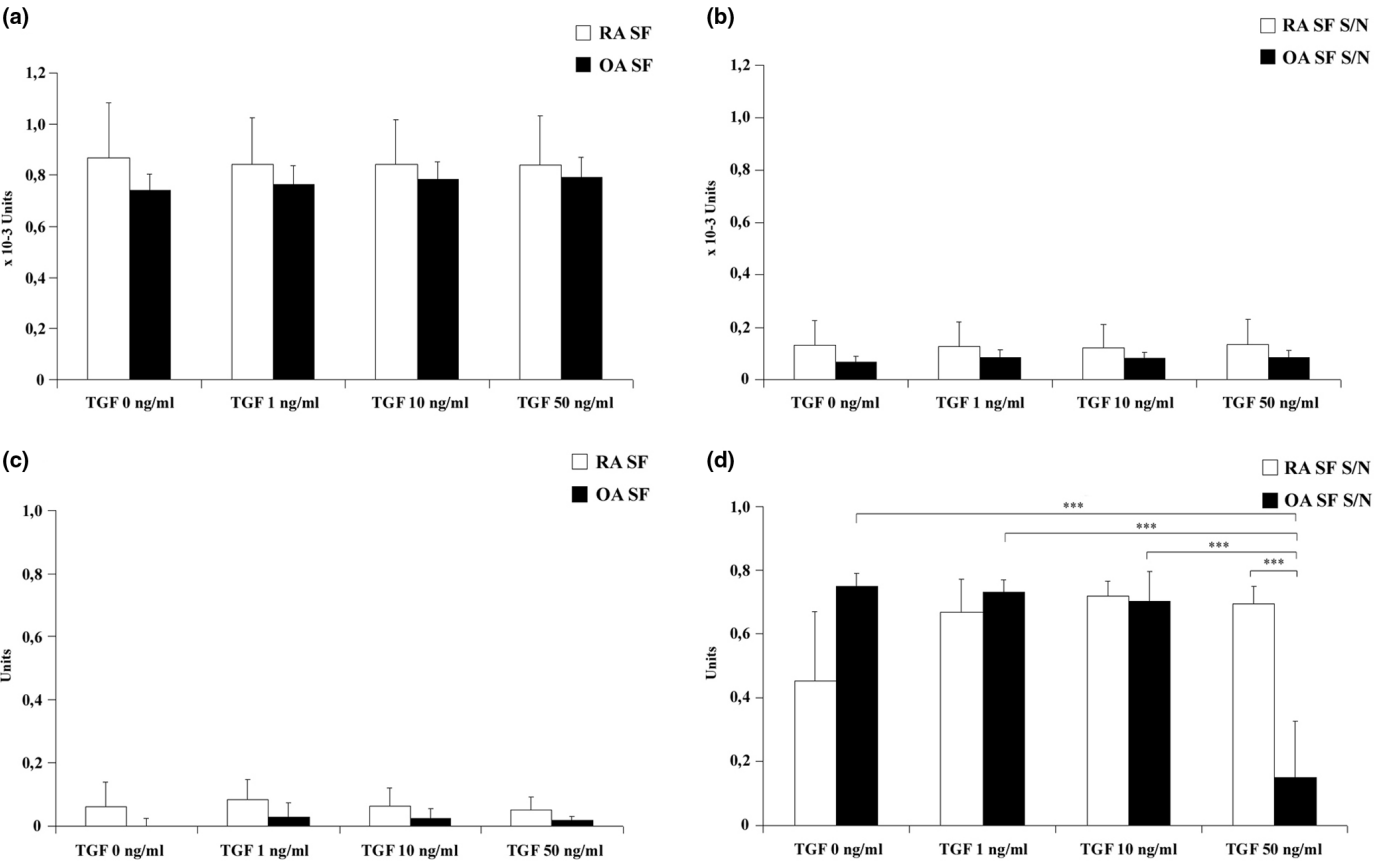
Enzyme activities of synovial fibroblast samples of rheumatoid arthritis and osteoarthritis patients after TGF-β_1 _treatment. Synovial fibroblasts (SFs) from patients with rheumatoid arthritis (RA) (n = 6) or osteoarthritis (OA) (n = 6) were cultured in the presence or absence of tumor growth factor beta 1 (TGF) for 24 hours. β-D-*N*-acetyl-glucosaminidase (NAG) and β-D-glucuronidase (GusB) activities were determined in cell lysates and the corresponding supernatants (S/N): **(a) **NAG in cell lysate, **(b) **NAG in supernatant, **(c) **GusB in cell lysate and **(d) **GusB in supernatant. Most NAG activity was found inside the SFs, while GusB was predominantly secreted into the supernatant. Data shown as mean ± standard error mean. *P *< 0.01 ***.

Since the influence of cytokines on gene expression was minor with the exception of TGF-β_1_, we measured whether TGF-β_1 _treatment had an effect on NAG or GusB activities in both SF lysates and in the SF supernatants. We found that most NAG activity was detected inside the cells (Figure 7a) and showed no significant changes under the effect of TGFβ_1 _either in the cell lysates or in the supernatant (Figures 7a, b). In contrast, minimal GusB activity was found to be associated with SFs and most GusB activity was found in the 24-hour supernatant of the cells (Figure 7c, d).

Although we did not detect any change in GusB activity in SF lysates, 50 ng/ml TGF-β_1 _treatment resulted in a significant decrease of secreted enzyme activity in the supernatant (Figure 7d).

### Detection of synovial fibroblast-derived microvesicles and microvesicle-associated GusB activity

**Figure 8:**
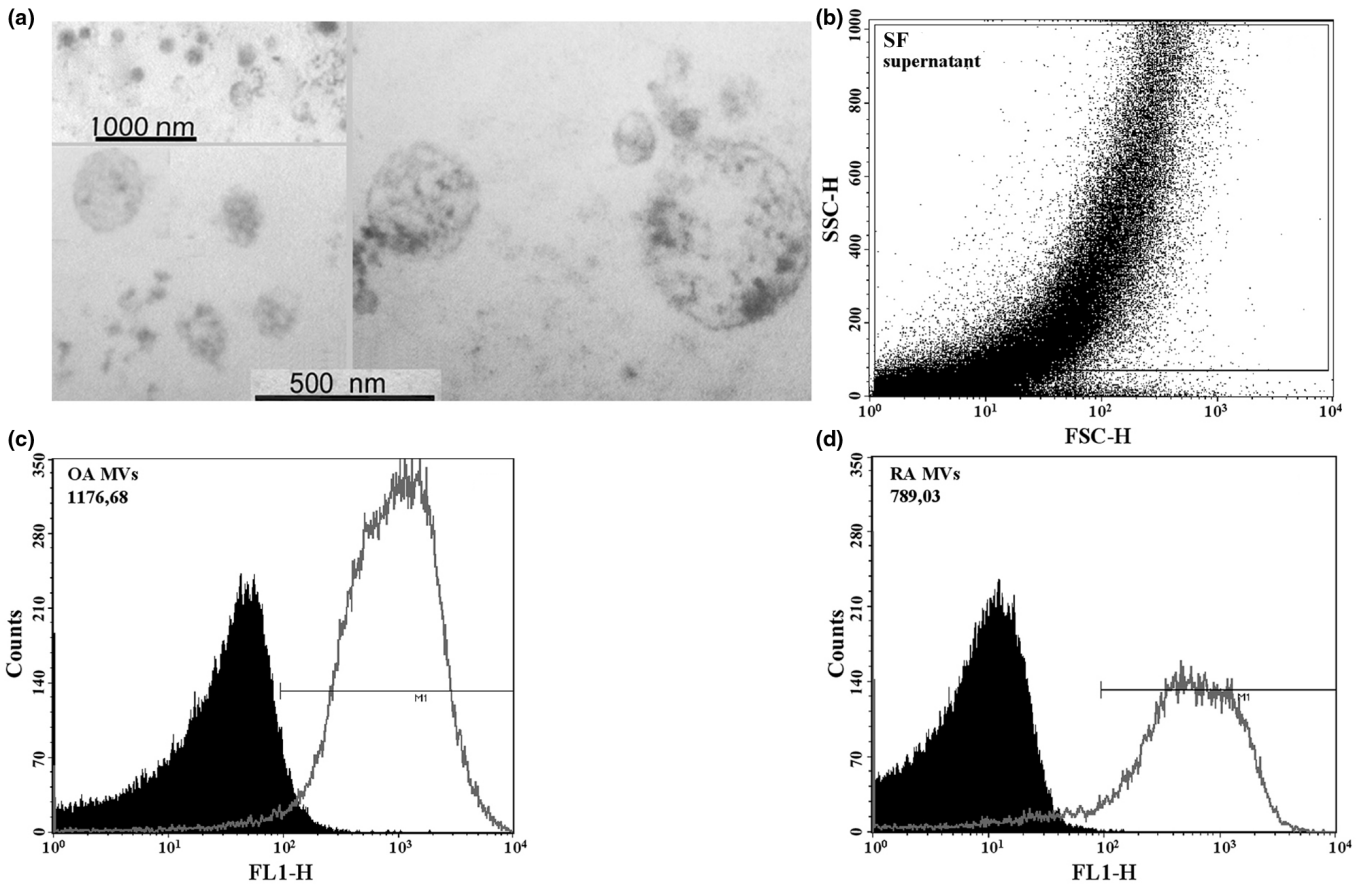
Detection of synovial fibroblast-derived microvesicles and microvesicle-associated GusB activity. **(a) **Synovial fibroblast (SF)-derived microvesicles (MVs) were isolated from serum-free 24-hour fibroblast supernatants by centrifugation and subsequent ultracentrifugation at 100,000 × *g*. Electron micrographs show different MVs varying in size and morphology. The dominant microvesicle type appears to be ectosome (diameter between 100 and 800 nm). **(b) **Flow cytometric scatter plots of 24-hour supernatants of SFs with cell-derived microvesicles. SSC-H (side scatter), FSC-H (forward scatter). **(c), (d) **Histogram plots show that the majority of rheumatoid arthritis (RA) and osteoarthritis (OA) SF-derived microvesicles are β-D-glucuronidase (GusB)-positive when stained with a lipophilic fluorogenic substrate. OA synovial fibroblast-derived MVs are characterized by stronger mean fluorescence intensity values than those derived from RA SFs. FL1-H (histogram of the green fluorescence).

To determine whether predominant glycosidases of SFs were also present in MVs, we tested the GusB and NAG activity associated with MVs in SF supernatants, SFl and serum samples of RA and OA patients using a lipophilic fluorogenic substrate. While we could not detect GusB activity associated with SFl-derived and serum-derived MVs, GusB activity was found to be associated with MVs in the supernatants of SFs of both RA and OA patients (Figure 8). The OA SF-derived MVs showed stronger GusB activity as compared with SF-derived MVs from RA patients. We could not detect NAG activity in synovial fibroblast-derived MVs using the fluorogenic NAG substrate.

## Discussion

While numerous studies have characterized the role of fibroblast-derived proteases in cartilage destruction [19-23], during the past decades surprisingly little attention has been paid to the activity of glycosidases in rheumatology. The few studies from the 1970s that reported elevated levels of glycosidases in joint diseases [24-26] were hardly followed by reports on glycosidases until recently. We earlier demonstrated the ability of exoglycosidases to degrade hyaline cartilage [5]. Popko and colleagues [27-31] and Shikhman and colleagues [32] reported high hexosaminidase activity in the joints of patients with rheumatologic diseases, and Li and colleagues have recently shown an increased heparanase activity in RA SFl and tissue [33]. The synovial glycosidase gene expression pattern has not yet been described, however, and it also remained unclear whether the gene expression of glycosidases in SFs was regulated by inflammatory cytokines.

We found a robust gene expression of the glycosidase-like Hc-gp 39 in the SMs, and in particular in SFs, of both RA and OA patients. The strikingly elevated Hc-gp 39 expression in SFs as compared with the SMs may be explained either by inhibition of its expression within the synovium or by upregulation of it by factors during *in vitro *growth of fibroblasts.

According to our data, hexosaminidase is the glycosidase with the highest expression and activity in the joints. This is in accordance with the findings of previous studies [29].

In the present study we show that SFs appear to be major sources of this enzyme in the SMs as they are characterized by strong expression of both HexA and HexB genes. Hexosaminidase A is composed of both alpha and beta chains, whereas hexosaminidase B is a homodimer of beta chains. The rare hexosaminidase S izoenzyme is composed from HexA – HexA gene products [34,35]. In this work we found a significantly higher expression of HexA compared with HexB in SFs and SM samples. This raises the intriguing possibility of intraarticular expression of the rare hexosaminidase S, responsible for degradation of sulfated glycosaminoglycans [36].

We hypothesize that even though SFs show relatively low expression of GusB, they might accumulate significant amounts of this lysosomal enzyme – some of which might be released by cell-derived MVs. This concept is supported by the GusB activity detected in cell lysates that was comparable with that detected in SM homogenates, and also by its association with cell-derived MVs.

The association of GusB activity with SF-derived MVs sheds light on a previously unrecognized localization of this enzyme.

Innate immunity plays a key role in the initiation of an immune response. Its germline encoded receptors such as Toll-like receptors (TLRs) detect danger signals. Functional TLR2 was reported in SFs of patients who had RA [37,38]. RA SFs, activated via TLR2, were suggested to contribute to arthritis development by secretion of chemokines. While exogenous TLR ligands have been investigated extensively, only few endogenous TLR ligands have so far been identified. These ligands include carbohydrate degradation products of the extracellular matrix (tetrasaccharides and hexasaccharides of hyaluronate and heparan sulphate) [39-41]. Interestingly, all known carbohydrate TLR ligands fall into the category of oligosaccharides generated by endoglycosidases, enzymes that cleave polysaccharide chains between nonterminal residues. The endoglycosidases that we have tested in the present study (Hyal1 and Spam1) showed minimal activity within the joints. Based on our results, therefore, it seems very likely that the carbohydrate degradation product ligands for TLRs are generated by exogenous (for example, microbial) endoglycosidases rather then SM-derived or SF-derived enzymes.

In line with earlier data, we found that hexosaminidase was the dominant exoglycosidase in the joints. Constitutive generation of cleavage products such as glucosamine by hexosaminidase may be part of the normal extracellular matrix/glycosaminoglycan turnover. Glucosamine has been recently shown to globally protect chondrocytes from the arthritogenic effects of IL-1β (by blocking the response in ~73% of IL-1β-stimulated genes) [42]. Glucosamine might therefore act primarily as an endogenous anti-inflammatory molecule within the joints. Under physiological conditions, hexosaminidase cleavage products may thus play a protective role and maintain tissue homeostasis, while this homeostatic balance may be shifted during microbial infections. In acute inflammation, frustrated phagocytosis and elevated intracellular free calcium level-induced secretion of lysosomal resident enzymes may result in significant release of further exoglycosidases by infiltrating cells (for example, monocytes and neutrophil granulocytes) [43] that might act in concert with SF-derived hexosaminidase. Upon the alternating action of certain exoglycosidases (hexosaminidase and glucuronidase), cartilage matrix degradation may dominate and lead to the release of glycosaminoglycans from the extracellular matrix.

One of the most striking findings of our study was that the regulation of gene expression of glycosidases and proteases by cytokines seems to be discordant. In sharp contrast to MMPs and other proteases, such as certain cathepsins – which have been reported to be highly inducible by proinflammatory cytokines [44-47] – we found that glycosidases were moderately downregulated by proinflammatory cytokines IL-1β, IL-17 and TNFα. The most pronounced cytokine effect was seen in the case of TGF-β_1_, which profoundly downregulated glycosidase expression in both RA and OA fibroblasts.

Transforming growth factor beta is found abundantly in the SM [48], and constitutive upregulation of the transforming growth factor beta pathway has been shown in RA SFs [49]. Transforming growth factor beta exerts both anti-inflammatory and proinflammatory actions, as exemplified by its ability to downregulate RANTES expression and by stimulating the synthesis of MMP-1 and IL-1 [50].

We hypothesize that the relatively stable expression and tight regulation control of synovial glycosidases are critical factors in the joints. Numerous cytokines exert complex regulatory mechanisms in RA. Our observation that glycosidases appear to be under negative control and are downregulated rather than stimulated by inflammatory cytokines may suggest that an enhanced expression of these enzymes could lead to severe and unforeseeable consequences. The extracellular matrix has been reported to serve as a repository of transforming growth factor beta and other growth factors of which the release is regulated via degradation of proteoglycans [51]. The long list of glycosaminoglycan binding proteins includes growth factors (like fibroblast growth factors, vascular endothelial growth factor, insulin-like growth factor-binding proteins), morphogens, enzymes, numerous cytokines and chemokines, interleukins, and so forth [52]. It can be hypothesized that a stringent control of the gene expression of glycosidases may prevent the synchronized release of the plethora of tissue-bound proteins.

Until recently, GusB has been regarded as a housekeeping gene in humans due to the absence of TATA box and high G+C contents within its putative promoter sequence [53]. Downregulation of GusB expression was demonstrated recently by the calcium ionophore A23187, the calcium ATPase inhibitor thapsigargin as well as by the calcium channel blocker verapamil in the human hepatoma cell line HepG2 [54]. The present study provides the first evidence that glycosidase gene expression (including the one of GusB) is regulated by human cytokines.

## Conclusions

Our data drive attention to the dominant negative regulation of a functional group of genes – glycosidases – by paramount cytokines in SFs that differs remarkably from regulation of proteases. The fact that we did not find significant differences between patients with RA and OA with respect to their glycosidase gene expression suggests a similar role and regulation for exoglycosidases in the two diseases. This hypothesis does not contradict these enzymes contributing significantly to cartilage degradation in both joint diseases if acting in concert with MMPs to deplete cartilage in glycosaminoglycans. Our data suggest that the earlier reported elevated glycosidase activities in RA joints were probably not due to enhanced gene expression of resident SFs, but rather resulted from enzyme release by cells (including infiltrating inflammatory cells) within the joints.

## Abbreviations

DMEM: Dulbecco's modified Eagle's medium; FCS: fetal calf serum; GusB: β-D-glucuronidase; HexA: hexosaminidase A subunit; HexB: hexosaminidase B subunit; Hyal1: hyaluronidase 1; IL: interleukin; MMP: matrix metalloproteinase; MV: microvesicle; NAG: β-D-*N*-acetyl-glucosaminidase; NOC-18: (*Z*)-1-(2-(2-aminoethyl)-*N*-(2-ammonioethyl) amino)diazen-1-ium-1,2-diolate diethylenetriamine; OA: osteoarthritis; PCR: polymerase chain reaction; RA: rheumatoid arthritis; RANTES: Regulated on Activation Normal T Cell Expressed and Secreted; RT: reverse transcriptase; SF: synovial fibroblast; SFl: synovial fluid; SM: synovial membrane; Spam1: sperm adhesion molecule 1; TGF-β_1_: tumor growth factor beta 1; TLR: Toll-like receptor; TNF: tumor necrosis factor.

## Competing interests

The authors declare that they have no competing interests.

## Authors' contributions

MP, GN, PN, AF and EIB participated in the design of the study. Experiments were performed by MP, PP, MCH, AK, KP and MM. GN, PG, TL, KT and KW contributed by providing human samples. Analysis of data was carried out by MP, GN, BG, AF and EIB. Intellectual contributions to the manuscript were provided by MP, GN, PG, TL, KT, KW, AF and EIB. All authors read and approved the final manuscript.

## Supplementary Material

Additional data file 1 An image file containing a figure demonstrating baseline glycosidase expression of SFs during passaging. The baseline glycosidase expression of SFs was tested at every second passage. There was no significant alteration of glycosidase gene expression from P1 to P9 passages either in OA (n = 6) or RA SFs (n = 5). Click here for file
