# Supplementary figures and images for: Gene expression and activity of cartilage degrading glycosidases in human rheumatoid arthritis and osteoarthritis synovial fibroblasts
Source: Arthritis Res Ther. 2009 May 14;11(3):R68. doi: 10.1186/ar2697 (PMC2714114; doi:10.1186/ar2697)

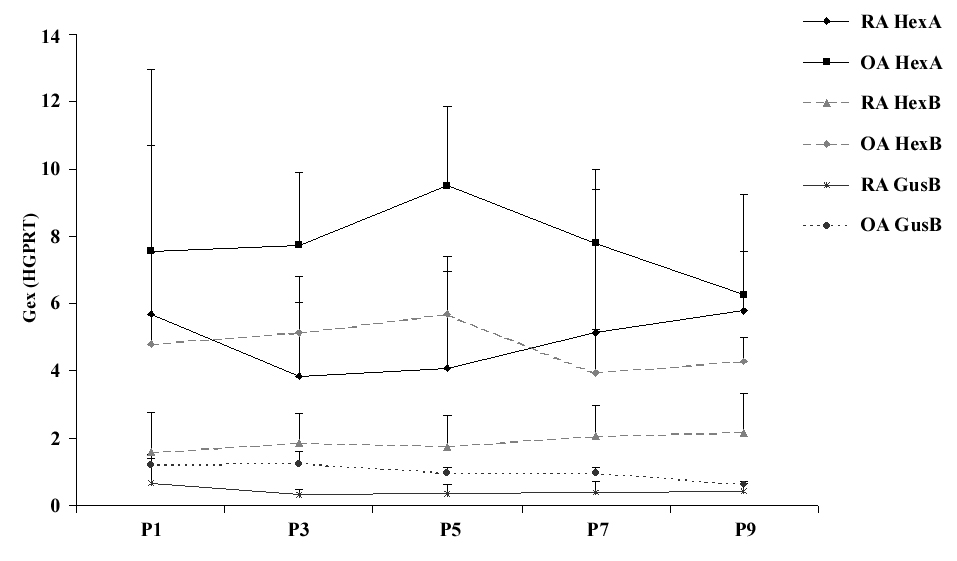

Supplement: Additional data file 1 — An image file containing a figure demonstrating baseline glycosidase expression of SFs during passaging. The baseline glycosidase expression of SFs was tested at every second passage. There was no significant alteration of glycosidase gene expression from P1 to P9 passages either in OA (n = 6) or RA SFs (n = 5). [file ar2697-S1.jpeg]
